# SARS-CoV-2 seroprevalence on the north coast of Peru: A cross-sectional study after the first wave

**DOI:** 10.1371/journal.pntd.0010794

**Published:** 2023-06-28

**Authors:** Luz M. Moyano, Angie K. Toledo, Jenny Chirinos, Percy Mc Quen Vilchez Barreto, Sofia Cavalcanti, Ricardo Gamboa, Jhon Ypanaque, Mauro Meza, Sheilla Noriega, Victor Herrera, Edgar Bazan, Alexandra Requena, Henry Silva, Harold Burgos, Franco León-Jimenez, Group of Neuroepidemiology and Science of Life of Peru

**Affiliations:** 1 Escuela de Medicina Humana, Universidad Cesar Vallejo, Piura, Piura, Peru; 2 Dirección Regional de Salud de Tumbes, Tumbes, Peru; 3 Unidad de Medicina Legal I Contralmirante Villar, Tumbes, Peru; 4 School of Public Health and Administration, Universidad Peruana Cayetano Heredia, Lima, Peru; 5 Centro de investigación para la preservación de la vida, Lima, Peru; 6 Center for Global Health, Universidad Peruana Cayetano Heredia, Tumbes, Peru; 7 Hospital Amistad Peru Corea Santa Rosa, II-2 Piura, Peru; 8 Escuela Profesional de Medicina Humana y Facultad de Ciencias en Salud de la Universidad Nacional de Tumbes, Tumbes, Peru; 9 Estrategia de enfermedades metaxénicas, Gobierno Regional de Tumbes, Tumbes, Peru; 10 Escuela de Medicina Humana, Universidad Norbert Wiener, Lima, Peru; University of Pittsburgh, UNITED STATES

## Abstract

**Background:**

Peru had the second-highest number of COVID-19 cases in Latin America. After the first wave, Peru registered more than 900,000 cases of COVID-19 and more than 36,000 confirmed deaths from the disease. Tumbes, a border area with poor sanitation and not enough water, had the fifth highest death rate. The cross-sectional analytic study aimed: a) to assess seroprevalence of COVID-19 after the first wave; b) to assess sociodemographic determinants and symptoms associated with a positive COVID-19 antibody lateral flow test.

**Methodology/principal findings:**

We performed this study between November 11th and November 30th, 2020, in an informal settlement in Tumbes. Individuals older than two years were invited to participate in a systematic random sample from one in every four households. Finger-prick blood samples were collected, and a census and symptom survey were applied. Within the chosen house, one adult over 18 years of age was chosen for a PCR-RT molecular test. Overall seroprevalence was 25.59%, adjusted seroprevalence was 24.82% (95%CI 22.49–27.25). Women had higher adjusted seroprevalence (28.03% vs 21.11%; 95% CI 24.83–31.41, *p = 0*.*002*). Symptoms as fever (PR 1.89: 95% CI 1.44–2.48, *p<0*.*001*), general discomfort (PR 1.67; 95% CI 1.23–2.26, *p = 0*.*001*), cough (PR 2.0; 95% CI 1.60–2.50, *p<0*.*001*), nasal congestion (PR 1.46; 95% CI 1.03–2.09, *p = 0*.*036*), respiratory distress (PR 1.64; 95% CI 1.04–2.56, *p = 0*.*031*), headache (PR 1.54; 95% CI 1.09–2.17, *p = 0*.*014*), anosmia (PR 1.78; 95% CI 1.01–3.14, *p = 0*.*046*) and ageusia (PR 2.31; 95% CI 1.48–3.61, *p<0*.*001*) were associated with a positive covid-19 antibody lateral flow test.

**Conclusions/significance:**

The COVID-19 transmission and distribution were highlighted by this cross-sectional study. The data will help the Ministry of Health improve its monitoring, surveillance, and monitoring of respiratory community sequelae in the future.

## Author summary

During the first wave, COVID-19 had devastating effects on developing countries like Peru, which reported more than 900,000 cases of the disease and more than 36,000 confirmed deaths from it. The informal settlements are counted in COVID-19 regional statistics, but they are very different from the rest of the region in terms of sanitation, access to water and sewage, housing conditions, and others. To make good decisions about public health, it is important to understand how the disease is spread in informal settlements. We did an observational, cross-sectional study in Puerto Pizarro, a remote port village in Tumbes, from November 11, 2020, to November 30, 2020, to assess the seroprevalence of COVID-19 after the first wave, as well as the sociodemographic factors and symptoms linked to a positive COVID-19 antibody lateral flow test. 1391 people older than 2 years old were given census and symptom questionnaires. They were also tested for the presence of anti-SARS-CoV-2 antibodies (IgG and IgM) in their blood. This study shows an adjusted seroprevalence of 24.82 percent (95% CI 22.49–27.25) posterior to the first wave of COVID-19 in Tumbes. Women had higher adjusted seroprevalence (28.03% vs. 21.11% [95% CI 24.83–31.41], p = 0.002). Extrapolating our adjusted prevalence of 24.82 per 100 persons to Tumbes (N = 251,541), 62,432 people were infected during the first wave, which is too high for adequate primary care in health institutions. Puerto Pizarro’s cause-specific mortality rate was 198.49 per 100,000 inhabitants (deaths documented between May and December 2020), which was higher than Tumbes’ up to December 2020 (137.6 per 100,000 inhabitants). The presence of mostly symptomatic patients at health care facilities, the limited use of social networks, the scarce availability of eHealth technologies in government institutions, unused emergency telephone numbers, and the fear of dying if referred to the hospital may have led to underreporting cases. Participation of the community in epidemiological surveillance strategies is critical to assisting the Ministry of Health in the future success of the establishment of an eHealth surveillance monitoring program.

## Introduction

In the last days of December 2019, the first cases of an unknown pneumonia were reported in the city of Wuhan, Hubei province, China [1,2]. After a few weeks of research, in January 2020, the Chinese Center for Disease Control and Prevention announced that they had found a new coronavirus named SARS-CoV-2 [3,4]. The World Health Organization (WHO) declared a pandemic of coronavirus disease (COVID-19), caused by the SARS-CoV-2 virus, on March 11, 2020 [5]. Even with Peru’s early confinement, COVID-19 wreaked havoc on developing world. [6]. The first case in Peru was reported in March 2020 in a 25-year-old European man [7]. The pandemic reveal a country with scarce health-care response and economic, political, and social instability [8]. Based on the 2017 census, 30 percent of the population does not have health insurance [9]. More than 70% of the population relied heavily on their daily work since they did not have a stable job, savings or health insurance that would allow them to comply with measures such as lockdown, so many government decisions were not feasible over time. Regional initiatives to determine the true spread of COVID-19 were performed in Iquitos [10], Lambayeque [11], and Lima [12], with prevalence rates ranging between 29.5/100 and 70/100 between June and August, 2020 using data from the CDC’s NOTIWEB notification program and the Ministry of Health’s (MINSA) SISCOVID software [13]. In Latin America, Peru had the second-highest number of COVID-19 cases between April 15, 2020, and December 31, 2020, when the first COVID-19 wave occurred [14,15]. Our country had registered more than 900,000 cases and more than 36,000 confirmed deaths of Covid-19 during the first wave. Tumbes reported at the end of 2020, 8.84% (22,239 /251,521pop) confirmed COVID-19 cases, and 724 confirmed COVID-19 deaths with a mortality rate of 287,8 per 100,000 individuals and fatality rates of 3.25% [16]. Previous to pandemic spread out, Tumbes region had 02 hospitals, 250 hospital beds between them, one small Oxygen station, 06/250 (2.40%) beds in ICU and, 20 oxygen-assistant beds, and four first level health care facilities with in-patient care [17].

National data underrepresent infection trends in informal settlements. Knowing about the disease’s spread and people’s immunological responses in these communities can help make public health policies that fit their requirements and use resources efficiently. Taking advantage of a large-scale community intervention in which the Tumbes Regional Government (GORE) and the Regional Directorate of Health (DIRESA) worked to find symptomatic cases through active house-to-house surveillance, we performed a cross-sectional study in the informal settlement of Puerto Pizarro to assess the SARS-CoV-2 seroprevalence, distribution by age group, and health determinants and symptoms associated with this respiratory condition in residents older than 2 years old.

## Materials and methods

### Ethic statement

The study protocol and consent forms were approved by the institutional review boards of Universidad Peruana Cayetano Heredia and the Regional Directorate of Health (DIRESA, by its Spanish acronym) of the Ministry of Health in Tumbes. The first one has an international registration in the Office for Human Research Protections effective until 03/21/2025. Also, it has a national registry accredited by the National Institute of Health of Peru, with indefinite validity.

To ensure comprehension, illiterate individuals were included by reading aloud the IC and survey. All participants signed the written informed consent (IC) in the presence of a witness (S1 Text). In the case of minors, the written informed consent was given by the minor and the parents or legal guardians (S2 Text).

### Area and study population

**Fig 1:**
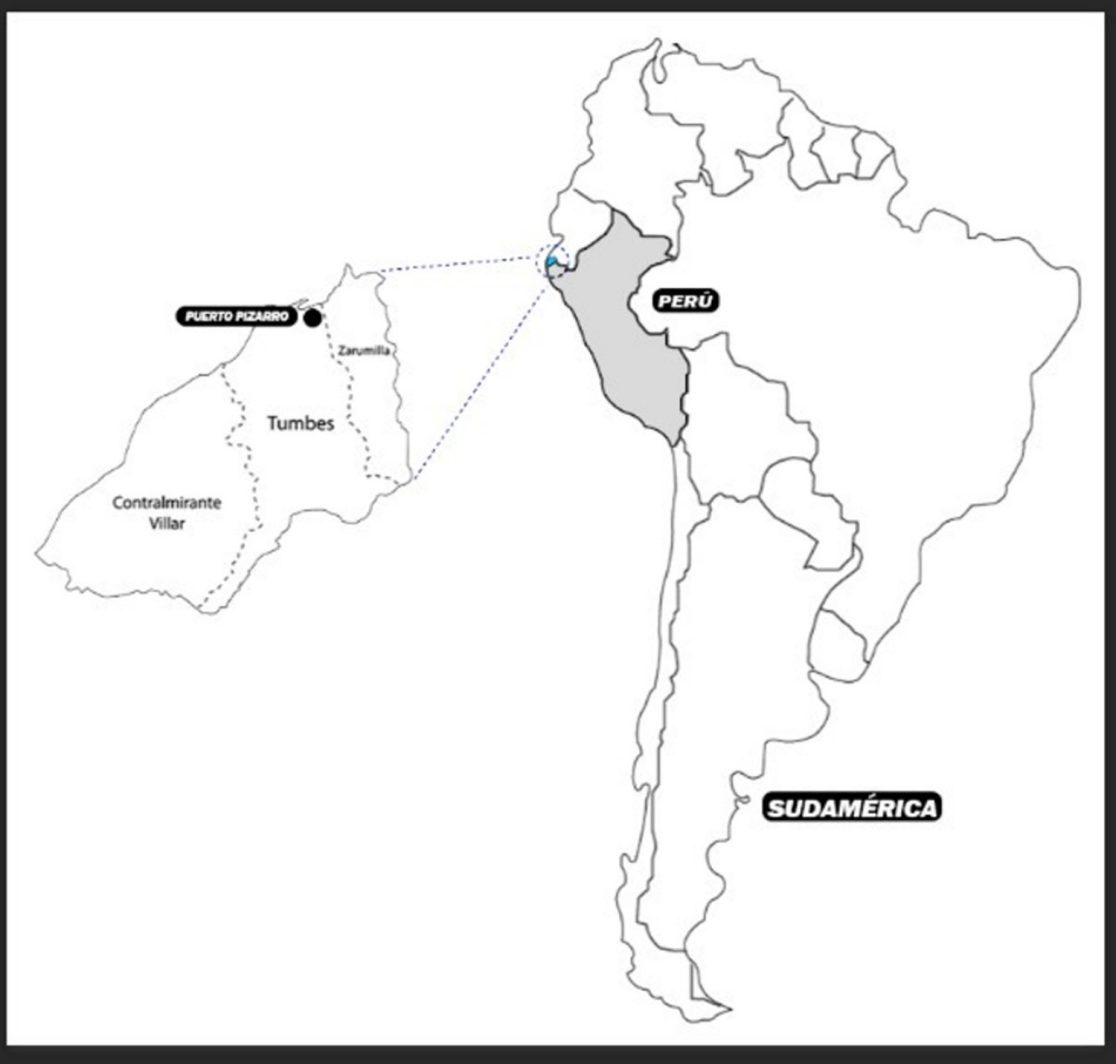
Map of Puerto Pizarro in Tumbes, Peru. “Reprinted from geogpsperu.com under a CC-BY license, with permission from Juan Pablo Suyo Pomalia [23].

With a population of 5908, Puerto Pizarro is an informal settlement at 13 kilometers north of Tumbes City (2017 census, INEI). The population is homogeneously mestizo and committed to fishing, trading, aquaculture, and tourism. Puerto Pizarro is a popular tourist destination that serves as the gateway to the Tumbes Mangrove Sanctuary [18,19] (Fig 1). 75% of the men in the community are artisanal fishermen, and 85% of the women are housewives [20]. Locals have limited access to water (1–2 hours per day), electricity, and sewage services. According to the 2017 national census, 20.3% Puerto Pizarro´s inhabitants have drinking water, and 11.2% have sewerage services (2017 census, INEI) [21].

A first level I-2 primary-care facility of 12 hours, open six days a week, is in Puerto Pizarro. Led by a chief and staffed by one newly licensed general practitioner, nurse, and midwife in one year of rural service. The health post has a waiting room, medical office, nurse room, delivery room, emergency room, and refrigeration storage for vaccines. SAMU (Mobile Emergency Care Service) provides ambulance coverage to transport COVID-19 patients with alarm signs to the hospital (10 km distance). During the first wave of the pandemic, the post had 3 gallons of oxygen available for a village that needed to provide services to 5,908 individuals. This population was chosen because it´s a representative informal settlement included in the COVID-19 statistics of Tumbes, which had the seventh highest mortality rate (133 per 100,000 inhabitants) during the first wave [22].

### Study design and sampling

A cross-sectional, observational study was done. For the systematic random sampling, dice were used to select the first house at random. Then, an interval was applied in which all individuals in one out of every four homes (n = 742) were asked to participate in the study.

### Recruitment

A nominal census with numbered houses organized by blocks was possible thanks to institutional collaboration between the regional directorate of Health-Tumbes and the Center for Global Health. To participate, individuals in selected households need to be older than 2 years old, sleep at least three nights a week in the village, and agree to sign the informed consent (IC). The recruitment and interviews were between November 11^th^ and November 30^th^, 2020. A total of 1391 individuals signed the IC and were included in the study [19]. Non-medical field workers (trained by a team of epidemiologists, LMMV, RGM, and PVB) performed a survey, took finger-prick blood samples, and explained the lateral flow test findings to all participants. According to the WHO, individuals were evaluated to identify clinical symptoms consistent with COVID-19 disease [24,25]. Individuals with respiratory symptoms were examined by a clinician from the study team and registered in the Notiweb and SISCOVID databases for follow-up by the health system. The Peruvian Ministry of Health’s COVID-19 Clinical Epidemiological Investigation format was used to identify symptoms over the last 14 days [26].

A census previously validated by the Center for Global Health in epidemiological population-based studies was applied [19,27]. Comorbidities defined as the presence of at least one of the following medical conditions were also identified: hypertension, diabetes, hepatic disease, renal disease, lung disease, asthma, obesity, neurological conditions, HIV, cancer, or tuberculosis [28]. An adult of legal age was selected from the same household, and an exploratory molecular test (RT-PCR) was performed to identify active cases.

### Lateral flow COVID-19 test

A lateral flow test (LFIA, lateral flow immunoassay) was used to detect the antibody positivity against SARS-CoV-2. STANDARD Q COVID-19 IgM/IgG (SD Biosensor, Republic of Korea) is a rapid chromatographic immunoassay for the qualitative detection of anti-SARS-CoV-2 nucleocapsid (N) protein antibodies and anti-SARS-CoV-2 spike RBD antibodies in human serum, plasma, or whole blood [29]. The manufacturer reports 99.03 percent sensitivity (102/103) and 98.65 percent specificity (219/222). Two trained reviewers (RG and VC) read the results, and a third reviewer resolved discrepancies (VH).

### Definitions and operationalization of variables

Seroprevalence of SARS-CoV-2 in Puerto Pizarro was defined as number of persons with positive result for IgG, IgM, or both by a lateral flow test divided by number of enrolled participants. Sex was a binary variable that can be female or male. The term "type of family" refers to a binary variable: single-family homes with a single-family nucleus and multi-family homes with two or more family nuclei. Rooms per person was defined as number of rooms in each house divided by number of persons in each house, then categorized in “1 room or less per person” and “more than 1 room per person”. There were two categories of water supply: non-shared connection (piped water directly to homes) and "shared connection" (which included neighboring supplies, common pipes, and water tanks). A binary variable titled "water storage" provides a "yes" or "no" response to the question "Do you need to store water for daytime use in buckets or containers?" The variable for hygienic services was separated into "latrines/don’t have" and "other and restrooms," which are defined as areas with a toilet, sink, network connection, and drainage. The variables associated with the water supply, water storage, and hygiene services were confirmed by visual inspection. When IgG, IgM, or both results from a lateral flow test have been presented, they were considered positive.

### Statistical analysis

We aimed to estimate the SARS-CoV-2 seroprevalence ratios in Puerto Pizarro performing descriptive statistics and binomial family generalized lineal models with a logarithmic link function, while controlling for other variables such as the participant’s age and gender. To account for household clustering, robust sandwich-type standard errors were utilized. Each variable was evaluated for inclusion in the final model using the log likelihood ratio. We report confidence intervals of 95% and set the level of statistical significance at p<0.05. Seroprevalence was adjusted for the test’s reported sensitivity (99.03%) and specificity (98.65%). Stata v 17.0 was used to perform the statistical analysis (College Station, Texas 77845, USA).

## Results

A total of 1916/5908 individuals older than 2 years were invited to participate, 1391/1916 (72.5%) signed an IC. Female participants were 53.3% (742/1,391). The overall participants’ mean age was 29±19 years. MINSA stratification mean ages were: 2 to 11 years (mean 6.72±2.81), 12 to 17 years (mean 14.40±1.65), 18 to 29 years (mean 23.71±3.47), 30 to 59 years (mean 42.66±8.18) and older than 60 years (mean 68.73±7.29). Most people lived in single families 1322/1391 (95.9%) and had a median of four rooms and four inhabitants per home. A large proportion of participants 1287/1391 (92.5%) had access to public potable water for a few hours (01–02 hours per day) and 1109/1391 (79.7%) had access to hygienic services.

### Seroprevalence

Anti-SARS-CoV-2 antibodies were detected in 356/1391 (25.5%) participants: 264 IgG reactive, 78 IgM/IgG reactive, and 14 IgM reactive. After adjusting for sensitivity (99.03%) and specificity (98.65%), the calculated adjusted seroprevalence was 24.82% (95%CI 22.49–27.25).

### Sociodemographic characteristics

Women had a slightly higher adjusted seroprevalence compared to men (28.03, 95% CI [24.83–31.41]) vs 21.11, 95% CI [18.03–24.45], *p = 0*.*005*) and had water storage (25.31, 95% CI [22.97–27.76] vs 17.58, 95% CI [10.40–26.98], *p* = 0.034) were associate to seropositivity. No other demographic characteristics were associated with SARS-CoV-2 lateral flow test seropositivity (Table 1). We conducted a logistic regression test to assess the association between the use of 4 water supplies (piped water directly to homes, neighboring supplies, common pipes, and water tanks) and risk of seroprevalence, but found no statistically significant differences were observed between the groups (p = 0.12).

**Table 1:** Seroprevalence adjusted for participant characteristics and associated factors.

Sociodemographic Characteristic	Total	Seropositive		Adjusted seroprevalence	PR* (95%CI)	p-value
	(N = 1391) n (%)	(N = 356) n (%)				
**Sex**						
Female	742 (53.34)	213	59.83	28.03 (24.83–31.41)	Ref.	
Male	649 (46.66)	143	40.17	21.11 (18.03–24.45)	0.76 (0.63–0.91)	**0.002**
**Type of family**						
Single family	1322 (95.04)	343	96.35	25.19 (22.87–27.62)	Ref.	
Multi-family	69 (4.96)	13	3.65	17.39 (9.32–28.41)	0.82 (0.60–1.12)	0.217
**Rooms per person†**						
≤1	967 (72.06)	217	70.68	21.59 (18.94–24.42)	Ref.	
>1	375 (27.94)	90	29.32	23.19 (18.85–27.95)	0.96 (0.78–1.18)	0.681
**Water supply**						
non-shared connection	1287 (92.52)	338	94.94	25.49 (23.12–27.96)	Ref.	
shared connection	104 (7.48)	18	5.06	16.35 (9.82–24.88)	0.84 (0.71–1.11)	0.309
**Water storage**						
No	91 (6.54)	17	4.78	17.58 (10.40–26.98)	Ref.	
Yes	1300 (93.46)	339	95.22	25.31 (22.97–27.76)	1.37 (1.02–1.83)	**0.034**
**Hygienic services**						
Other and restroom	1109 (79.73)	294	82.58	25.79 (23.24–28.47)	Ref.	
Latrine / don’t have	262 (5.97)	62	17.42	22.90 (17.95–28.47)	0.94 (0.83–1.08)	0.391

Adjusted seroprevalence estimated considering the sensitivity (99·03%) and specificity (98·65%) of the diagnostic test.

95%IC: 95% confidence interval. P-values were calculated using glm, fam (bin) link (log).

* Adjusted for house

† Missing values

**Fig 2:**
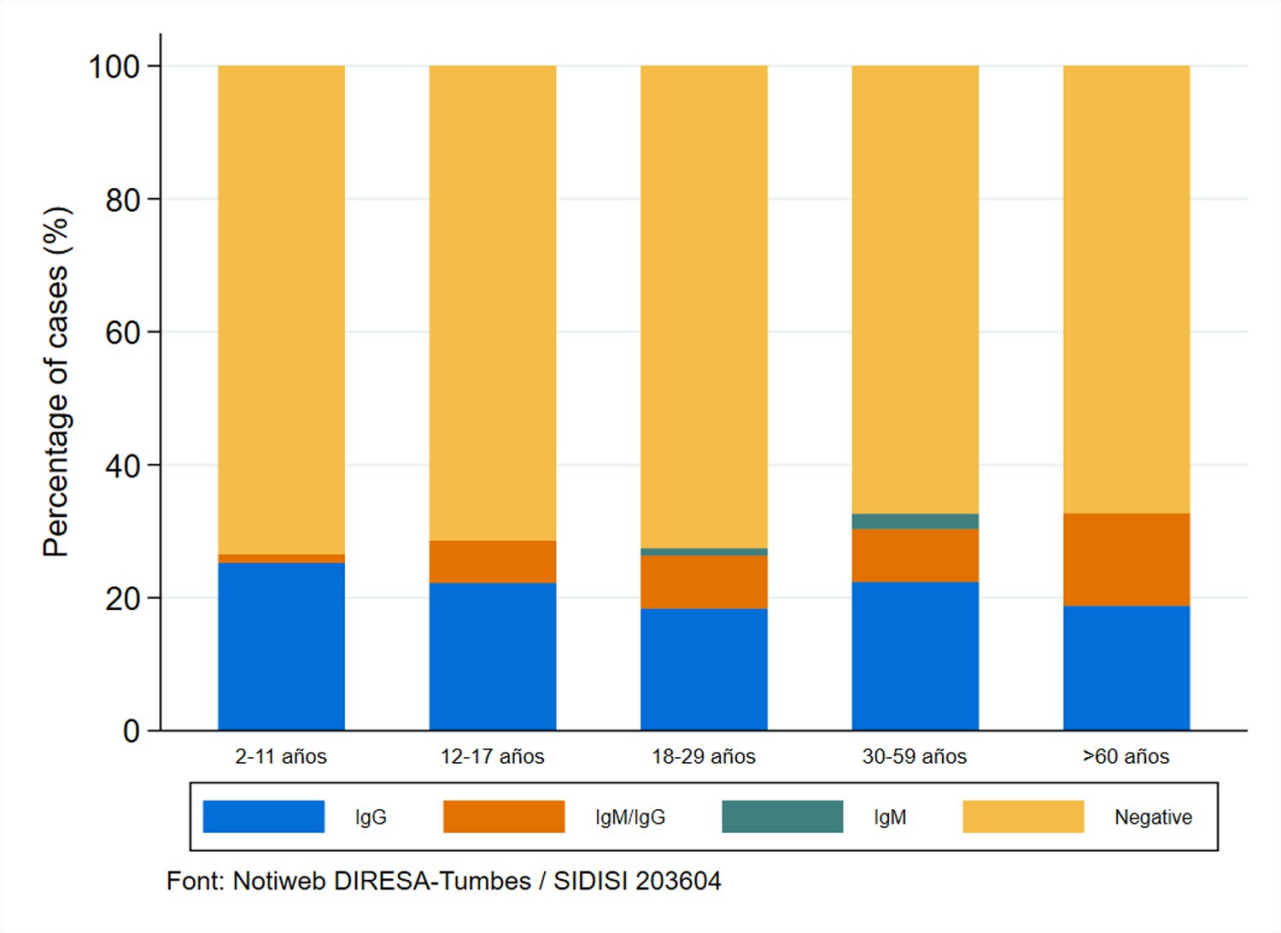
Seronegative and seropositivity in Puerto Pizarro.

As illustrated in Fig 2, the age groups with the highest prevalence of IgG seropositive were from 2 to 11 years (25.25%), from 30 to 59 years (22.40%), and from 12 to 17 years (22.22%).

**Fig 3:**
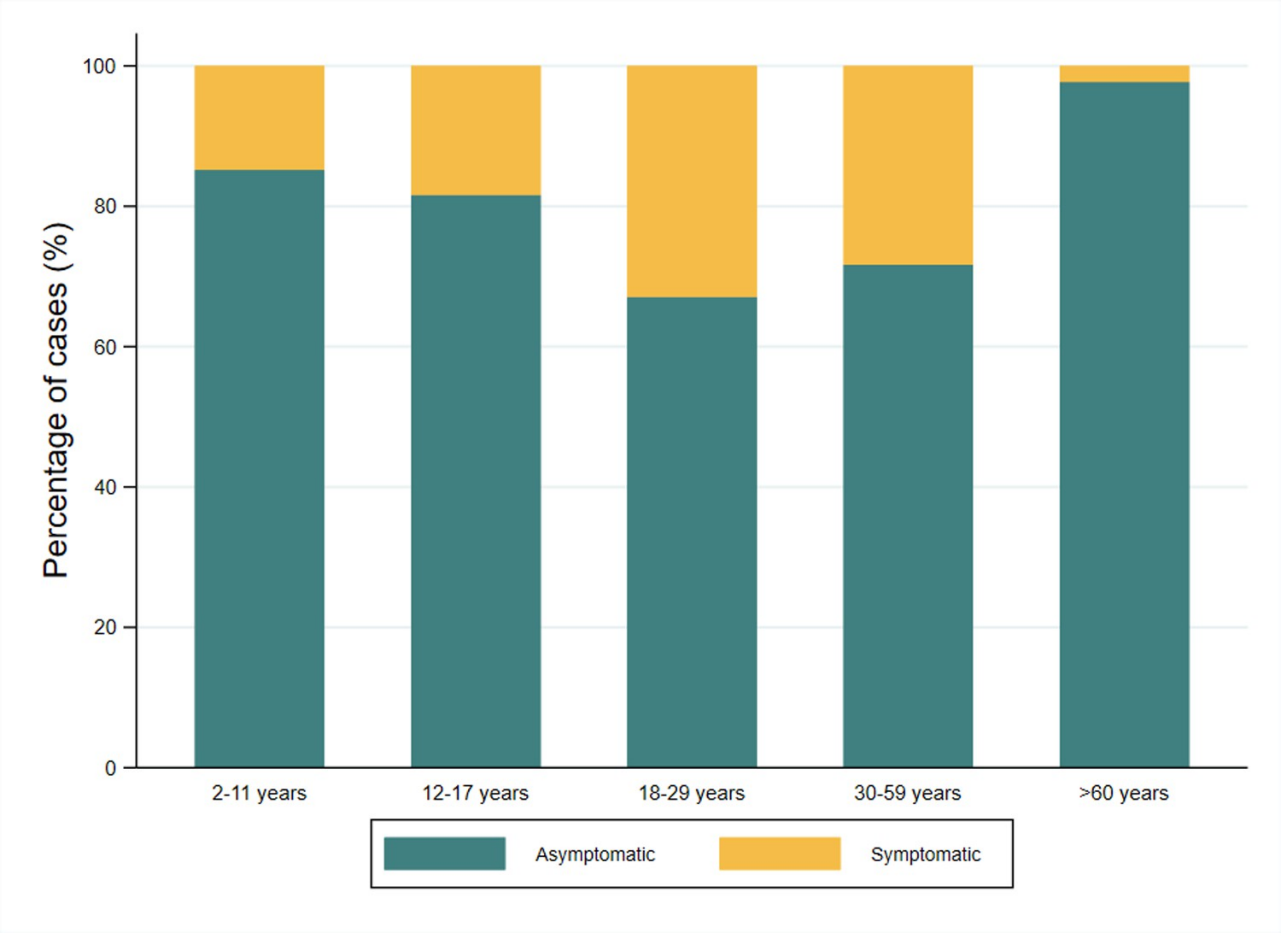
Symptomatic and asymptomatic cases in the seropositive population.

The RT-PCR test revealed no active cases, and no deaths were reported among the participants. On the other hand, more than 60% of seropositive patients were asymptomatic in all age groups (Fig 3).

In a bivariate analysis, numerous symptoms such as fever (PR: 1.89, 95% CI [1.44–2.48]), general discomfort (PR: 1.67, 95% CI [1.23–2.26], p<0.001), cough (PR: 2.00, 95% CI [1.60–2.50]), nasal congestion (PR: 1.46, 95% CI [1.03–2.09]), respiratory distress (PR: 1.64, 95% CI [1.04–2.56]), anosmia (PR: 1.78, 95% CI [1.01–3.14]) and ageusia (PR: 2.31, 95% CI [1.48–3.61]) were associated with SARS-CoV-2 seropositivity. Cough was the unique symptom associated with seropositivity in a multiple analysis (PR: 1.78, 95% CI [1.24–2.57]) (Table 2). There were no comorbidities associated with seropositivity.

**Table 2:** Self-reported symptoms between seropositive and seronegative participants.

Covid-19 symptoms	Positives		Negatives		PR (95% CI)	p-value*	PRA (95% CI)	p-value**
	n = 356		n = 1035					
	Yes	no	yes	No				
Fever	34	322	42	993	1.89 (1.44–2.48)	<0.001	1.29 (0.84–1.98)	0.254
General Discomfort	28	328	42	993	1.67 (1.23–2.26)	0.001	-	-
Cough	53	303	63	972	2.00 (1.60–2.50)	<0.001	1.78 (1.24–2.57)	0.002
Sore Throat	39	317	126	909	0.95 (0.71–1.27)	0.716	-	-
nasal congestion	21	335	38	997	1.46 (1.03–2.09)	0.036	1.08 (0.67–1.75)	0.742
Respiratory Distress	18	338	12	1023	1.64 (1.04–2.56)	0.031	0.99 (0.53–1.88)	0.999
Diarrhea	3	353	12	1023	0.81 (0.29–2.23)	0.676	-	-
Sickness	3	353	4	1031	1.73 (0.73–4.10)	0.209	-	-
Headache	22	334	37	998	1.54 (1.09–2.17)	0.014	-	-
Irritability	2	354	5	1030	1.15 (0.35–3.74)	0.811	-	-
Muscle pain	4	352	6	1029	1.62 (0.75–3.48)	0.215	-	-
Abdominal pain	1	355	2	1033	1.34 (0.27–6.69)	0.716	-	-
Chest pain	4	352	0	1035	-	-	-	-
Join pain	2	354	0	1035	-	-	-	-
Anosmia	7	349	9	1026	1.78 (1.01–3.14)	0.046	-	-
Ageusia	9	347	7	1028	2.31 (1.48–3.61)	<0.001	1.57 (0.78–3.15)	0.202

* p-value were calculated with glm, fam(bin) link(log)

** p-value were calculated glm, fam(bin) link(log), adjusted for anosmia, fever, cough, respiratory distress and nasal congestion

## Discussion

In Tumbes, the first wave of COVID-19 was between March 16, 2020, and the first days of December 2020. The present cross-sectional serological survey was performed eight months after the first case of COVID-19 detected in Tumbes, between November 11th and November 30th, 2020. Serology was performed during the epidemiological weeks (EW) 45–48, posterior to the first official peak of cases and mortality in Tumbes (S1 Fig). On December 31, 2020, Tumbes reported 22,239 confirmed COVID-19 cases with a positivity proportion of 8.8% (251,521 pop.) and 724 confirmed COVID-19 deaths with a cause-specific mortality rate of 309.71 per 100,000 inhabitants [30]. Therefore, this study will help to understand how the first wave of COVID-19 affected this population’s serology. Puerto Pizarro had an adjusted seroprevalence of 24.82% (95% CI 22.49–27.25), whereas the seroprevalence in other regions of Peru’s interior ranged between 10.2 and 70% [10–12].

Seropositivity in the present study was slightly higher compared to similar studies conducted in other developing settings such as Nairobi (22.7% [95% CI: 18.0–27.7]) or Palestine (24.0% [95% CI: 21.7–26.5]) [31,32], but lower to that found in rural communities such as Iquitos-Peru (70.0% [95% CI: 67.0–73.4]), Manaus-Brazil (76% [95% CI: 67.0–98.0]), and Ecuador (45%) [33–35]. The high seroprevalence could be associated with the behaviors of people regardless of whether they belong to a developed or developing country, and perhaps to the development time of the other studies, which was between June and August 2020 (the middle of the wave), and our study was almost at the end of the first wave. After the first 8 months of the pandemic, the Netherlands, a developed country, had 19.5% SARS-CoV-2 seroprevalence [36]. The factors associated with exposure to the virus were no social distancing and a high number and duration of contacts [36]. An epidemiological study in Lima, Peru found that a gradual increase in the overcrowding index was associated with a higher seroprevalence of COVID-19 (aPR = 1.99 [95% CI 1.41–2.81, p<0.001] in the fourth quartile) [12]. However, the number of rooms and family members were not associated with positive seroprevalence in our study.

Artisanal fishing is the main work of the community members in Puerto Pizarro. 75% of the men in the village work in this informal, but unionized, occupation [20]. Small-scale fishing requires people to leave their homes every day to sell the marine products, increasing the risk of Sars-CoV-2 virus being contagious person-to-person and spreading COVID-19 during the first wave, which is consistent with what was observed in Lima, Peru, where a gradual decrease in socioeconomic status was associated with a higher seroprevalence (aPR = 3.41 [95% CI: 1.90–6.12, p<0.001] in low socioeconomic status) [12].

The lack of adequate accessibility to potable water (available 1 or 2 hours per day), the use of tanker trucks, commercial and unlicensed suppliers, and informal arrangements with neighbors [37,38] which increase human contact on a daily basis, are possible explanations for water storage have been linked to increased positive seroprevalence (p = 0.034), decreasing the effectiveness of COVID-19 prevention and control measures. Also, this may suggest that individuals who store water are at a higher risk of experiencing an unstable water supply, which could lead to difficulties in maintaining household cleanliness and hygiene. Consequently, this could elevate the chances of transmission and seroprevalence. Our study found no association between positive seroprevalence and water supply [10,11,39] even though 20.3% of people have access to drinking water service and 11.2% have access to sewerage service [21].

When adjusted for participant characteristics, women had higher adjusted seroprevalence compared to men (213/356 vs 143/356 [28.01% vs 21.18%], p = 0.005). Similar studies reported no association between sex and seroprevalence during the first wave of COVID-19 [40–43]. While other studies indicated that men were more likely to contract SARS-CoV-2 [44–47]. Numerous studies cite the propensity of men to work outside the home, their greater contribution to pre-existing diseases, and their higher risk of risky behaviors such as smoking as potential explanations for this association. [44–47]. One possible theory about how SARS-CoV-2 spreads is that the mothers’ market shopping increased the risk of getting sick in both rural and urban areas, considering that 85% of the women in the region are housewives [20]. In fact, according to an epidemiological report done by the Peruvian Ministry of Health, up to 86% of merchants in Peruvian markets had COVID-19 as of June 2, 2020 [48].

In order to restrict the spread of SARS-CoV-2, it is crucial to determine which age group is continually exposed to the virus. In our based-population study, the age groups with the highest prevalence of IgG seropositive were from 2 to 11 years (25.25%), from 30 to 59 years (22.40%), and from 12 to 17 years (22.22%) (Fig 2), assuming that many asymptomatic individuals were not detected when they acquired the virus. In Iquitos (Jungle, Peru) study findings, a higher seroprevalence was observed in the age group younger than 12 years old [42], in Saudi Arabia, the seroprevalence of SARS-CoV-2 was higher in the 1 to 18-year-old group [40]; in both studies most of the seropositive children were asymptomatic. Children, despite being asymptomatic, are just as susceptible to COVID-19 as adults. The first possible explanation is that SARS-CoV-2 uses ACE2 as a viral receptor, whose tissue distribution and binding capacity may be lower in children compared to adults [49], and the second is a higher number of B lymphocytes, T lymphocytes, and natural killer cells proposed as an immunological mechanism for children to resist infection [50,51]. The evidence reveals that the viral load does not differ considerably between symptomatic and asymptomatic individuals; therefore, asymptomatic individuals represent a source of the transmission of the virus [52,53]. Although some hypotheses suggest that children are not the primary carriers of the infection, they are infected in their homes through contact with infected adults [54].

Fever, sore throat, and cough were the most frequently reported symptoms in COVID-19 in earlier research in Lima-Peru, occurring in 40% to 50% of seropositive symptomatic patients [55]. Dysosmia (PR = 1.69), chest pain (PR = 1.49), back pain (PR = 1.45), cough (PR = 1.44), fever (PR = 1.41), and general malaise (PR = 1.27) were also shown to be linked with an increased prevalence of SARS-CoV-2 seropositivity in Lambayeque’s study [11]. Our findings corroborated those seen in national and international reports [56,57]; cough was the single symptom associated with a positive COVID-19 lateral flow test, suggesting that the disease’s behavior may differ between different places.

In our study from all seropositive individuals more than 60.0% were asymptomatic across all age groups (see Fig 3). This exceeds previously reported evidence suggesting that asymptomatic transmission of SARS-CoV-2 may account for between 30–59% of all infections [58]. Also, infected people can be asymptomatic carriers, complicating their identification by health systems and being the principal viral transmitter inside their homes [59,60]. In this context, community screening is required to determine the true dynamics of infection transmission and to adjust control measures through population-based tactics such as active search for suspected cases (door-to-door registration) and early case confirmation with antigen rapid testing.

Our research had a limitation due to the small size of the sample, and there could be differences in characteristics between the sample size, level of representativeness of broader population and categorizations in the survey, making the results generalizable to similar settings. Also, even though the surveys were applied with a standardized census used by the Regional Directorate of Health and the Center for Global Health at Tumbes Facilities, some of the categories of the survey, such as type of family, water supply, and water storage, did not accurately reflect the diversity of responses, leading to biased or inaccurate results. The study’s RT-PCR results revealed no active infections or deaths at the time of the study. Also, because we didn’t do RT-PCR molecular tests on people who didn’t have antibodies, we couldn’t figure out how many people with no symptoms and no antibodies were infected. Tumbes had a regional laboratory equipped for molecular detection of the virus with personnel trained in molecular biology and epidemiology techniques, but the lack of materials for molecular testing limited us to performing tests to identify active disease on the first wave in our case [8].

The mortality rate associated with COVID-19 was not the target of this research, but the Regional Directory of Health-Tumbes reported 10 of 5908 deaths in Puerto Pizarro caused by COVID-19, and the cause-specific mortality rate in this community was 169.26 per 100,000 inhabitants (deaths were recorded between May and December 2020), which was higher if we compare it against Tumbes’ cause-specific mortality rate up to December 2020 (137.6 per 100,000 inhabitants). At the end of December 2020, the highest accumulated mortality rates in Peru were from the regions of Ica (184 per 100,000 inhabitants), Callao (175.2 per 100,000 inhabitants), and Moquegua (160.8 per 100,000 inhabitants) [22].

Extrapolating the adjusted prevalence from our study (24.82 per 100 people) to the region of Tumbes (N = 251,541), about 62,432 individuals were infected during the first wave, too high for optimal primary care in health facilities. The number of COVID-19 cases in the region exceeded what has been reported in regional health bulletins [61]. Multiple variables may have contributed to the underreporting: the presence of primarily symptomatic patients at health care facilities; inadequate use of social networks; scarce eHealth tools in government facilities; unused emergency telephone numbers; and the fear of dying if referred to the hospital. In future studies, home-made antigenic rapid tests for early viruses using smartphone technology could be a plausible solution to be explored.

Some key behaviors related with the risk of COVID-19 infection, such as rigorous home isolation, appropriate use of masks and recent close contact with positive individuals, were not analyzed and may be investigated in future research in Tumbes. Diaz-Velez et al, in Lambayeque´s study, demonstrated that individuals who complied with rigorous home isolation had a 20% lower likelihood of testing positive for SARS-CoV-2 (PR = 0.80). The odds of testing positive went up by 60% (PR = 1.60) in contact with patients with acute respiratory illness, 51% (PR = 1.51) in contact with a confirmed case in the past 14 days, and 26% (PR = 1.26) in visits to the market [11].

## Conclusions

This study shows a high prevalence of SARS-CoV-2 antibodies after the first wave of COVID-19 in Tumbes, which can provide an estimation of the infection attack rate during this period. A multidisciplinary effort must be made to understand the development of the disease and the behavior of the communities around COVID-19. So far, there is an urgent need to understand the real situation of COVID-19 in vulnerable populations to improve surveillance and control programs.

## Supporting information

S1 Data Excel spreadsheet containing, in separate sheets, the underlying data used for the statistical analysis and description of variables. (XLSX)

S1 Text Adult informed consent for study participation approved by the institutional review boards of Universidad Peruana Cayetano Heredia. (PDF)

S2 Text Parent-signed informed consent for minor study participation approved by the institutional review boards of Universidad Peruana Cayetano Heredia. (PDF)

S1 Fig COVID-19 behavior in Tumbes as of January 1, 2022 (SE52), according to the Situation Room of the Tumbes Regional Health Directorate, based on data from Notiweb, SINADEF, and NetLab2. Complete information on confirmed cases, cumulative incidence rates, death, and others may be found at https://www.diresatumbes.gob.pe/images/COVID-19/SALASITUACIONAL/2022/enero/SALA_COVID-19_-_Ene-01.pdf.</SI_Caption >
(TIF)
