# Supplementary material for: SARS-CoV-2 seroprevalence on the north coast of Peru: A cross-sectional study after the first wave
Source: PLoS Negl Trop Dis. 2023 Jun 28;17(6):e0010794. doi: 10.1371/journal.pntd.0010794 (PMC10335682; doi:10.1371/journal.pntd.0010794)
Supplement: S1 Text — (PDF) [file pntd.0010794.s002.pdf]

INFORMED CONSENT TO PARTICIPATE IN A RESEARCH STUDY

(Adults)	
Study Title :	**Prevalence of COVID-19 Associated with Dengue and Malaria in Rural and Urban Communities of the Tumbes Region: Prevalence of COVID-19, Tumbes**
Researcher (s) :	**Gamboa Moran Ricardo, Moyano Vidal Luz Maria, Vilchez Barreto Percy Mcquen**
Institution :	**Universidad Peruana Cayetano Heredia**

Purpose of the study:

We are inviting you to participate in a study to evaluate the number of COVID-19, dengue, and malaria cases in your community, with the aim of identifying the spread of this new disease and the coexistence of COVID-19 with other regionally relevant diseases such as dengue and malaria. This study is being conducted by researchers from the Universidad Peruana Cayetano Heredia, the Regional Health Directorate, and the National University of Tumbes. If you do not wish to participate, your decision will be respected, and you will still have the opportunity to undergo the rapid tests or nasal swabs required for the study at your nearest healthcare center. Before accepting your participation, we will provide you with all the information so that you can make an informed decision. You are encouraged to ask any questions you may have, and we will gladly respond. When you have no further doubts and have made the decision to participate, please sign this document.

Procedures:

If you decide to participate in this study, it will take approximately 30 minutes to complete the following activities:

1. A rapid test and/or nasal swab will be conducted to detect COVID-19. The rapid test will detect antibodies against the virus, and the nasal swab will measure the viral load (this will be randomly done on a family member). The personnel responsible for sample collection will wear personal protective equipment throughout, including face shields, N95 masks or similar, disposable gowns, disposable gloves, hand sanitizer, and/or 70% alcohol. They will also maintain a distance of 2 meters during the interview. These measures will be followed during all sample collections.
2. A blood sample will be requested to rule out dengue, and a rapid test will be conducted to rule out malaria. The personnel responsible for sample collection will also wear personal protective equipment.
3. An interview will be conducted lasting approximately 10 minutes, where you will be asked about your personal information and symptoms related to COVID-19, dengue, and malaria.

Risks:

This study does not pose any health risks. You may experience some pain and redness at the needle insertion site, but it will pass quickly. If the puncture causes any discomfort, the study doctor will attend to you immediately. There is a possibility that some of the questions may generate some discomfort, but you are free to answer them or not.

Benefits:

You will benefit from undergoing a rapid test and/or a respiratory tract swab to detect or rule out COVID-19, as well as the screening for vector-borne diseases such as dengue and malaria, all of which are provided at no cost. We will provide you with important information on how to **PROTECT YOURSELF and AVOID Dengue, Malaria, and COVID-19**. Those who test positive for any of these diseases will be referred to the Health Center in their jurisdiction to have access to medication and follow-up.

COMITÉ INSTITUCIONAL DE ÉTICA UNIVERSIDAD PERUANA CAYETANO HEREDIA DUICT CIE
APROBADO
F. APROBACIÓN 08 11

INFORMED CONSENT TO PARTICIPATE IN A RESEARCH STUDY

**(Adults)**	
Study Title :	**Prevalence of COVID-19 Associated with Dengue and Malaria in Rural and Urban Communities of the Tumbes Region: Prevalence of COVID-19, Tumbes**
Researcher (s) :	**Gamboa Moran Ricardo, Moyano Vidal Luz Maria, Vilchez Barreto Percy Mcquen**
Institution :	**Universidad Peruana Cayetano Heredia**

Costs and compensation

The costs associated with the application of the different COVID-19, dengue, and malaria detection tests will be covered by DIRESA-TUMBES and will not incur any expenses for you or your family members. You should not pay anything to participate in the study. Likewise, you will not receive any financial or other incentives.

Confidenciality:

All data collected in this study will be stored and kept confidential in a private location, accessible only to study personnel. Your information will be stored using alphanumeric codes and not your name. Your data will not be shared with any individuals outside of the study without your permission, except for the organizations responsible for monitoring the safety of all research participants. If the results of this study are published, the names of the participants will not be disclosed.

FUTURE USE OF INFORMATION

We intend to store the data collected in this research for a period of 5 years. These data may be used for future research related to the detection, prevention, and/or containment of the new diseases COVID-19, dengue, and malaria. The stored data will not contain names or any other personal information; they will only be identifiable by codes to respect participant confidentiality.

If you do not wish for the data collected in this research to be stored or used for future purposes, you can still participate in the study. In that case, once the research is completed, your data will be deleted.

Prior to the use of your data in any future research project, that project will require approval from an Institutional Research Ethics Committee.

I authorize the storage of my data for a period of 5 years for future use in other research projects. (After this period of time, the data will be deleted).
YES (   )   NO (   )

Participants Rights:

It is important for you to know that if you choose not to participate in the study, this decision will not affect your comprehensive medical care at your healthcare facility. Additionally, if you are already part of the study, you have the right to withdraw your participation at any time. In either case, your regular medical care will not be negatively affected, and you can continue to visit the healthcare center for your usual care.

APROBADO
F. APROBACIÓN: __ / __ /

08 11

INFORMED CONSENT TO PARTICIPATE IN A RESEARCH STUDY

**(Adults)**	
Study Title :	**Prevalence of COVID-19 Associated with Dengue and Malaria in Rural and Urban Communities of the Tumbes Region: Prevalence of COVID-19, Tumbes**
Researcher (s) :	**Gamboa Moran Ricardo, Moyano Vidal Luz Maria, Vilchez Barreto Percy Mcquen**
Institution :	**Universidad Peruana Cayetano Heredia**

You can ask questions about the research to the study personnel at any time. You can contact **Dr. Luz María Moyano Vidal at mobile number 973822863** or **Biol. Ricardo Gamboa Morán at mobile number 997531821.** If you have any doubts about your rights in the study, you can communicate with the President of the Institutional Ethics Committee at Universidad Cayetano.

If you have questions about the ethical aspects of the study or believe that you have been treated unfairly, you can contact Dr. Frine Samalvides Cuba, President of the Institutional Research Ethics Committee at Universidad Peruana Cayetano Heredia, at telephone number 01-3190000 extension 201355 or via email at duict.cieh@oficinas-upch.pe.

A copy of this informed consent will be provided to you..

DECLARATION AND/OR CONSENT

I have read the informed consent form and have been given the opportunity to discuss and ask questions about it. By signing this document, I voluntarily agree to participate in this study, understanding that I have the right to withdraw at any time without any negative impact on my regular medical care thereafter.

Participant's name Signature Date

Witness's name (in the case of illiterates) Signature Date

Researcher's name Signature Date

COMITÉ INSTITUCIONAL DE ÉTICA UNIVERSIDAD PERUANA CAYETANO HEREDIA DUICT CIE
APR 08 11
F. APROBACIÓN: / /
