# Supplementary figures and images for: SARS-CoV-2 seroprevalence on the north coast of Peru: A cross-sectional study after the first wave
Source: PLoS Negl Trop Dis. 2023 Jun 28;17(6):e0010794. doi: 10.1371/journal.pntd.0010794 (PMC10335682; doi:10.1371/journal.pntd.0010794)

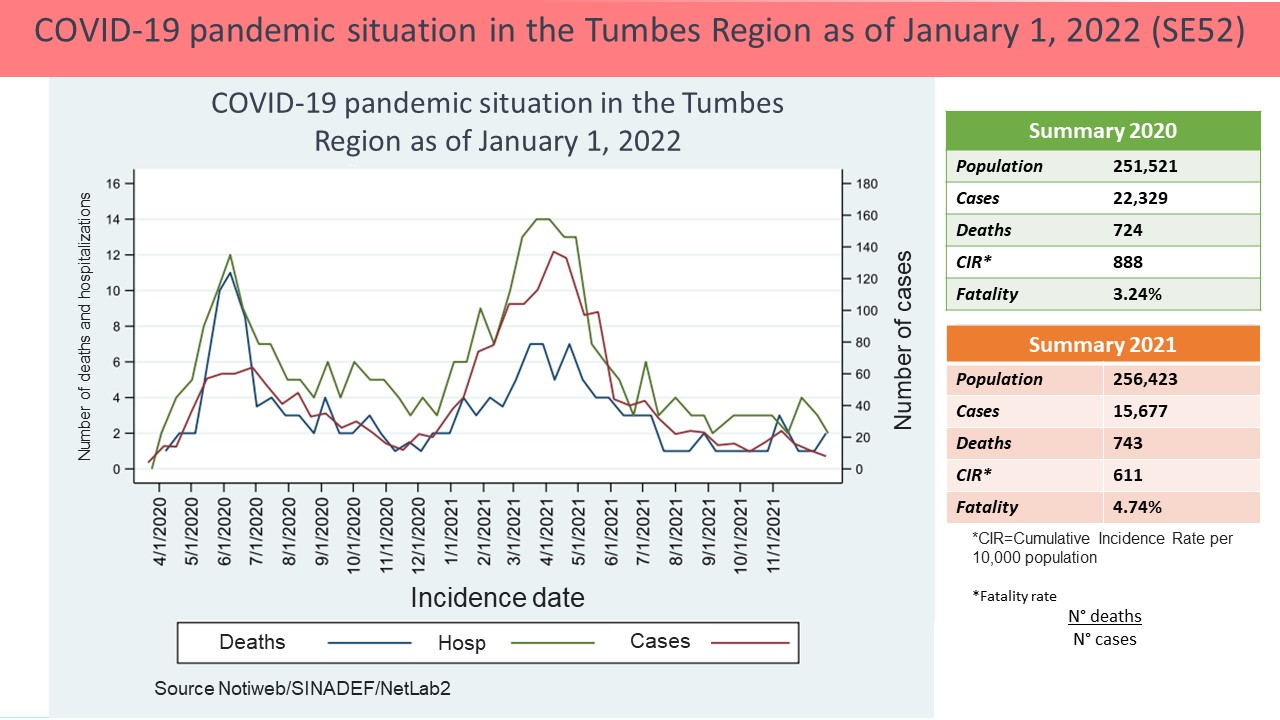

Supplement: S1 Fig — > (TIF) [file pntd.0010794.s004.tif]
